# Optimization of the Spatial Position of the Vibration Acceleration Sensor and the Method of Determining Limit Values in the Diagnostics of Combustion Engine Injection System

**DOI:** 10.3390/s26061981

**Published:** 2026-03-22

**Authors:** Jan Monieta, Lech Władysław Kasyk

**Affiliations:** Maritime University of Szczecin, 70-500 Szczecin, Poland; l.kasyk@pm.szczecin.pl

**Keywords:** internal combustion engine, fuel injection system, damage progression, device and method, diagnosis

## Abstract

A new procedure for diagnosing damage to the fuel injection system of marine engines, along with vibration acceleration signal symptoms, is explored with a related built, developed, and tested measuring system. This work fills an important gap given the current lack of a scientific solution to this problem. A vibration acceleration signal sensor, mounted on a holder elaborated on by the authors, is positioned on the injection pipe between the injection pump and the injector. The output signals from the sensor are sent to an acquisition and analysis system, which is used for processing the signals in the time, amplitude, frequency, and time–frequency domains. Experimental choices, using multiple parameters for a given signal analysis field, are based on the location of the optimal sensor, the direction of the sensor mounting, and the selection of a cumulative diagnostic symptom. The vibration acceleration signals recorded along the injection pipe are found to have the strongest magnitude. This article compares diagnostic values from these signals with previously determined upper and lower limits. As a result, the tested fuel injection system is classified as either able or disabled, using unparalleled tolerance ranges given for both the upper and lower limits. The values of the limits are determined based on the average value for an ability state plus or minus three times the standard error of this mean, which has not been reported in the literature previously. Multiple regression models are developed that relate identified symptoms to the state features of the fuel injection system. In addition, artificial neural networks and machine learning are used to detect developing damage. The probability of correctly classifying the states of the diagnostic parameters is 0.467, alongside a diagnostic accuracy of ≤±4%, with the network correctly classifying the state when the testing accuracy is at least 70.0%.

## 1. Introduction

During the operation of self-ignition engines (SIEs), some damage to metal components may go undetected, which leads to secondary or significant failures [1]. At present, diagnostic systems used in floating vessels, such as ships, primarily rely on thermal signals, but they may alert the crew too late. In a previous study, Monieta [2] applied selected parameters to the accompanying and working processes signals for the diagnostics of the development of failure in selected elements of marine SIEs. Methods such as decimation, windowing in the time domain, amplitude analysis, time–frequency analysis, wavelet transforms, image color measures, and machine learning have been used for damage classification. For these methods, diagnostic symptoms and their determined limit values were identified and selected.

Fractographic studies have shown that cracks that initiate nozzle damage occur in a thin-walled groove filled with fuel [3,4]. A limitation for previous methods when measuring pressure waveforms was the lifting of the zero point on the sensor’s contact with the fuel due to an electrical charge leak [5]. In addition, it has been discovered that many vibration displacement measurement methods interfere with engine operations due to the added mass on the intermediate element, which introduces inertial forces that affect the movement of the nozzle needle [5]. Noninvasive diagnostic methods are shown to overcome this problem. To date, however, the measurements of the vibration acceleration signals have shown large variation for the diagnostic parameters across different domains at specific measurement locations on internal combustion engines or test benches [2].

Injector damage accounts for a significant proportion of disability states and has a major impact on the operation of SIEs, causing disabilities, knocking, reduced power output, and even engine damage [6]. The relationships with the technical states have been inconclusive, with extremes observed. Accurate estimation of the injection parameters from monitored vibrations is currently a method for detecting and diagnosing damage in the fuel injection system.

The impact of fractures in steel on acoustic emission signals was previously examined by Miesovich et al. [7]. Fatigue fracturing exhibits distinct stages of development [7], i.e., structural changes, burst initiation, and stabilized propagation until the termination of the fracturing process. Detecting crack initiation is particularly challenging due to its stochastic nature [1,7]. To improve the detectability of engine damage, a time–frequency analysis was developed to account for the variability of vibration signals over time. Typical methods included short-term Fourier transform, fractional Fourier transform, Gabor–Wigner transform, linear canonical transform, and Stockwell transform [8].

Technical and modeling developments, along with increasing environmental regulations, have spurred the need for more efficient and reliable methods of diagnosing SIEs. The focus is on diagnosing damage as it develops and identifying the causes of secondary damage. Research by Krogerus et al. [9] argued that, as the injector opening pressure required to meet increasingly stringent regulations on noise, smoke, and exhaust toxicity increases, the fuel injection system becomes the most unreliable component. An overview of the diagnostic methods for this system and the types of damage, especially in the development phase, were provided. Damage initiation in SIEs alters the frequency spectrum. Therefore, the choice of sensor mounting affects the correct diagnosis [10].

The fracturing of SIE shafts and injector nozzles has been assessed by determining the number of stress cycles or the necessary operating time for fracture growth from an assumed initial size to a final size [11]. To prevent such failures, the condition of the injectors is monitored to detect developing damage [12]. Cylinder pressure and acoustic emission signals, in either the time domain or synchronized with the crankshaft rotation angle and frequency, have been used [6,12]. It has been shown that cylinder pressure signals in the time domain are poor diagnostic parameters, while lower-order spectrum measures are more reliable [6,12].

Other research by Bejger [13] tested cracked and detached nozzles in marine engine injector use. This author used acoustic emission signals to perform a diagnostic of the fuel injection system. Fractures in the mechanical elements of ICEs dissipate vibration energy [14]. Additionally, a report by Czech et al. [15,16] used noninvasive vibroacoustic methods to diagnose SIE damage. These authors used wavelet analysis and artificial neural networks to analyze the signals. Advanced signal processing techniques have been used to detect faulty injectors based on specific symptoms [17]. The classifiers were trained under normal injector operating conditions and with research data for comparison.

Work by Asi [18] conducted research on the damage to the nozzle of an SIE injector of a car made from 18CrNi8 steel. The nozzle failed after approximately 400 h of operation. Photographic imaging, chemical composition analyses, microhardness measurements, and metallographic analyses were performed. This work concluded that the damage was caused by cavitation wear inside the injector nozzle, followed by fatigue fracturing. There are known methods for diagnosing the fuel injection system by recording changes in the fuel pressure waveform in relation to the camshaft rotation angle and comparing them with a reference waveform [6]. According to ISO 22547:2021 [19], other parameters include tests for high-pressure marine pumps and auxiliary devices.

Artificial intelligence has also been used for injector diagnostics by other researchers [2,14,20,21]. For example, the study of Vaz et al. [21] used a deep neural network to predict fuel atomization characteristics. Their innovative analysis indicated that factors, such as the pressure waveform in the dual-fuel marine injection system, the number of nozzle holes, the geometry of the outlet holes, and the volume of the nozzle bag, significantly affect the atomization process. These authors argued that artificial intelligence offers an alternative to time-consuming computational fluid dynamics simulations and experimental methods. There is a lack of available, high-quality datasets representing different types of injector damage under representative conditions. Research by An et al. [22] reported using dual injectors across two nozzle hole diameters, but they did not measure the nozzle geometry or the fuel mass flow rates through individual holes or nozzles. A dynamic model of an object was developed using a fuzzy inference system, which considered various damage scenarios to train a neural network [23]. The method’s accuracy was verified using a benchmark simulator.

The innovation described in a previous article [24] outlines a device and a method for diagnosing the condition of injector valves using engine vibrations. The engine control system unit included several modules, including injector fault diagnosis. A patent [25] offers a device for frequency spectrum analysis that evaluates the operating parameters of an ICE, which is synchronized with the engine crankshaft position. A signal processing device performs a frequency spectrum analysis of the output signal from a knock sensor using the discrete Fourier transform and fast Fourier transform techniques. Moreover, Wigner–Ville vibration distribution processing was applied to the vibration signals, using time–frequency maps, which were correlated with the fuel injection parameters [26]. Clearly, estimating the injection parameters from external monitoring of vibrations is a useful method for detecting damage to the injection system.

The Welch statistics, short-time Fourier transform, Wigner–Ville distribution, and Choi–Williams conversion were applied to study the vibrations generated by an underloaded engine. A damaged injector component results in higher-frequency elements from 10 to 25 kHz. When comparing the vibration responses of functional and faulty injectors, the root-mean-square (RMS) value and kurtosis of the faulty injectors increased by 12.9% and 20.6%, respectively [27].

Combining a vibration sensor with a cylinder pressure sensor improves the diagnostic capabilities of the fuel injection system of the marine SIEs [2,6,12,21]. In multihole injectors, differences in geometric and material structures lead to variations in spray characteristics. Research has been conducted that uses the principles of similarity and transformation theory for injectors with different numbers of holes and high-pressure waves in injector spaces ranging from 100 to 300 MPa [28].

Medeiros et al. [29] published innovative research on vibration signal time series and acoustic pressure levels from an SIE injector, which identifies parameters in the processed signals using a wavelet analysis. The authors found a correlation between the signal frequency measures and the injector wear. A study by Neumann et al. [30] used signals from a vibration sensor to determine the timing of the nozzle needle displacement and the dosing start in the time domain, and the fuel delivery cutoff of the injection pump. Noninvasive diagnostic methods for various faults in the fuel injection systems were presented, which used vibration time histories and mathematical simulations of the engine operations. However, these methods require precise and well-calibrated mathematical models.

In other work, Dudziński and Kluczyk [31] presented the results of multistage measurements of the injector technical conditions. Injectors were modeled to obtain resonance frequencies and tests were performed to record the vibration parameters relating to the condition of the injector. In addition, Kluczyk et al. [32] reported cases of fatigue fractures in the nozzles and injector springs, noting that the nozzle collided with the piston. Self-ignition engines often have injectors located under the valve covers, which limits the placement of direct sensors. Furthermore, the study by Kaźmierczak and Wróbel [33] employed short-time and fast Fourier transforms with parametric and nonparametric time windows to diagnose the pump injectors through a vibroacoustic signal analysis. This study determined the relationship between the condition of the common rail injectors and the leak parameters to develop methods that identify particular leaks and locate injector damage [33]. The authors presented a device for implementing these methods and determining leak parameters for the fuel injection system.

Current research aims to reduce the time needed to diagnose electrohydraulic injectors, increase informativeness and accuracy, and develop methods for continuous monitoring of injector condition and damage prediction. This makes it difficult to identify leaks in the injection subsystem under extreme operating conditions of accumulator injectors [34].

The study by Mączak et al. [35] created innovative methods for diagnosing electromagnetic fuel injectors by recording the electrical current parameters in the engine controller. According to the authors, the applied method enables the detection of electrical and mechanical damage in the early stages, which prevents further damage to other engine systems.

In the technical diagnostics of ICEs, identifying the initial stages of wear and faults is often complicated. In the study of ICE vibrations, advanced signal analysis techniques are used to make accurate decisions regarding multiple diagnostic parameters [36]. For example, research has been conducted on a set of injectors used in biofuel feed systems that were damaged during operation [37]. The study found plastic deformation, channel obstruction, microfractures, erosion, and cavitation wear. For diagnosing injector valve damage, time wavelets and spectral analysis have been used [38]. However, it is unfortunate that basic signal processing methods still dominate in these areas, with quantitative time domain and time–frequency analyses not employed; only illustrative graphs for different states have been provided [2,13,24,38]. Studies on new and operated objects have often been reported, which assume they are fully functional and disabled, respectively [12,13,17,29].

Although various methods have been developed to detect fractures in ICE components, despite the granting of patents [22,23], their effectiveness and implementation have been unsatisfactory [2,3,27,30,32,33]. Therefore, there is a need to estimate injector parameters from monitored vibrations [12,30,39,40] and apply methods for diagnosing, locating damages, and genezing (determining the causes of the observed state) damage in the fuel injection system. An alternative method is required for setting, checking, and adjusting the fuel injection processes, with a clear reference to limit values [2,34,38].

The current state of technology means that vibration acceleration sensors are mounted invasively using threaded connections [41] or using less-accurate noninvasive holders and methods [42]. In his doctoral thesis, Mazurak [42] presented a holder for mounting a sensor on the fuel pipe ferrule in a common rail injection system to diagnose using acoustic emission signals. In the corresponding article [6] and in other authors’ work [41], alternative methods for mounting a vibration sensor using different holders have been proposed, but the correlation with the input values was found to be relatively low. Thus, in most cases, sensors were still mounted invasively using threaded connections [38].

Previous studies, as stated earlier, often tested new and used components, assuming full ability and disability states, respectively. Only a few studies have estimated measurement errors, despite reporting results without the accompanying error estimates being unreliable [2,3,5,10]. Clearly, there is a need to estimate the injector parameters from monitored vibrations [39,40,43].

Although vibroacoustic analysis is promising, it is not fully optimized for identifying specific types of damage. Based on current knowledge, there is a research gap in the optimization of sensor locations, the scientific selection of useful diagnostic symptoms from among many parameters, and the accurate estimation of lower and upper limit values. This study aims to address this existing gap by introducing a scientific method for estimating injector parameters and detecting faults using vibration measurements, and locating any damage in the injection system.

## 2. Materials and Methods

### 2.1. Research Problem

Methods for diagnosing fuel injection systems with vibration acceleration signals are typically still at the research stage. In such experiments, the selection of diagnostic symptoms for the fuel injection systems is made using a set of measured parameters. The latter focuses on indicators of damage with a high diagnostic performance. This article explores the use of an innovative measuring system of this kind and discusses the selected diagnostic parameters of the vibration acceleration signals for detecting damaged elements in ship compression–ignition engines. The research problem of this article is formulated by analyzing selected multidimensional diagnostic parameters from vibration acceleration signals, from which it is possible to reliably assess the technical conditions of the fuel injection systems in marine SIEs at different stages of their operational lifetime.

The primary objective of this research is to design a device that optimally diagnoses a fuel injection system, particularly for detecting damage. The optimization criterion was the highest energy at different locations of the vibration acceleration sensor. Such a device applies to conventional, electronically controlled fuel injection systems of internal combustion engines in both transport and stationary configurations. This work also develops a method for determining the limit technical state (either ability or disability) of the fuel injection system by the use of the vibration parameters across various domains. Moreover, artificial intelligence tools are used to identify damage. The results accurately identify a faulty state, which is associated with a reduction in fuel oil consumption, less toxic emissions into the atmosphere, and lower noise levels from the combustion engines. This research is conducted under both laboratory conditions (active and passive–active experiments) and the natural operating conditions of marine vessels during port handling and shipyard works (passive and passive–active experiment).

### 2.2. Research Method

Accurate measurements are invaluable for diagnosing the fuel injectors of SIEs from vibration acceleration signal parameters. The outputs of the latter are processed in domains of time, amplitude, frequency, and wavelet transform in the time–frequency domains. They are then compared with previously determined limit values. Based on this comparison, the fuel injection system under examination is classified into one of the two technical states, i.e., ability or disability. Namely, it depends on whether the analyzed system resides within a certain range or not, respectively, which is based on predetermined upper and lower tolerance intervals of the state.

The vibration acceleration signals are measured at a sampling frequency of 100 kHz over at least three cycles. A set of values of a selected diagnostic parameter has its signal compared with the previously determined limit values, and then the examined fuel injection system is classified accordingly. The diagnostic symptoms are analyzed in the amplitude domain (interpeak values *a*_*p*-*p*_), frequency domain (average amplitude *H_aaver_* values and/or root-mean-square value, RMS, values in tertiary bands *H_arms_*, such as the nineteenth harmonic component, *H_aaver_*_19_, of the rotational frequency multiplied by 100), and the time–frequency domain (wavelet at approximation levels 1, 3, and/or 5).

The upper and lower limits determine the ability state, while the unfit state is defined by the criteria for values above or below these limits. The measurements that attain the technical condition of the fuel injection system require the same speeds (crankshaft or camshaft), fuel setting of the pump rack, and thermal states of the SIE or test bench. Otherwise, they must be corrected to standardized conditions for repeatability reasons [44].

The presented method uses *n* diagnostic parameters, each relating to the various characteristics of the fuel injection system’s technical state. Distinguishing it from existing evaluation methods, this procedure defines the grounds for the two-class scale that is used to evaluate the technical condition of the fuel injector. The technical state of the system is determined using an average uncertainty relative to the mean. Namely, limit values are calculated based on the mean value of a parameter for an ability state plus or minus three times the absolute arithmetic mean-square of the mean (AAMSM). The limit values are estimated with a 0.997 probability level for the calculated mean diagnostic results. The error fields are determined by subtracting and adding the AAMSM using the following formulas (based on information given in a patent [44]):

(1) $$P_{allv}=\bar{P_{a}}\;-3\sigma_{s}\left(P_{a}\right),$$

(2) $$P_{aulv}=\bar{P_{a}}+3\sigma_{s}\left(P_{a}\right),$$

where *P_allv_* is the lower limit of the diagnostic measurements, *P_aulv_* is the upper limit of the diagnostic measurements, ${\bar{P}}_{a}=\frac{1}{n}\sum_{i=1}^{i=n}P_{ai}$ is the mean value of the diagnostic measurements, $P_{ai}$ is the *i*th value of the diagnostic parameters, *n* is the number of measurements, and *σ_s_* is the calculated mean error of the mean (MEM) or, more precisely, the average square error of the arithmetic mean of the diagnostic parameter [5], which is 1/$\sqrt{n}$ smaller than the mean-square error of each measurement, i.e.,

(3) $$\sigma_{s}=\frac{\sigma }{\sqrt{n}}=\sqrt{\frac{1}{n(n-1)}\sum_{i=1}^{n}(P_{ai}-\bar{P_{a}}{)}^{2}},$$

where σ denotes the standard deviation. The expanded uncertainty indicates that 99.7% of the results, corresponding to 1 − 0.997, fall within the range $\bar{P_{a}}\;-3\sigma_{s}\left(P_{a}\right)$ to $\bar{P_{a}}\;+3\sigma_{s}\left(P_{a}\right)$. This means that, out of 1000 results, only 3 may fall outside the tolerance range.

The lower (*llv*) and upper (*ulv*) limits, which define the suitable and faulty states, are determined based on the tolerance and state features of the model injectors. An ability state is found when all the diagnostic symptom values are within the production and/or operational tolerances. Since engine operating conditions affect the diagnostic parameters, it is recommended to use an average load level of 58% [45].

**Figure 1:**
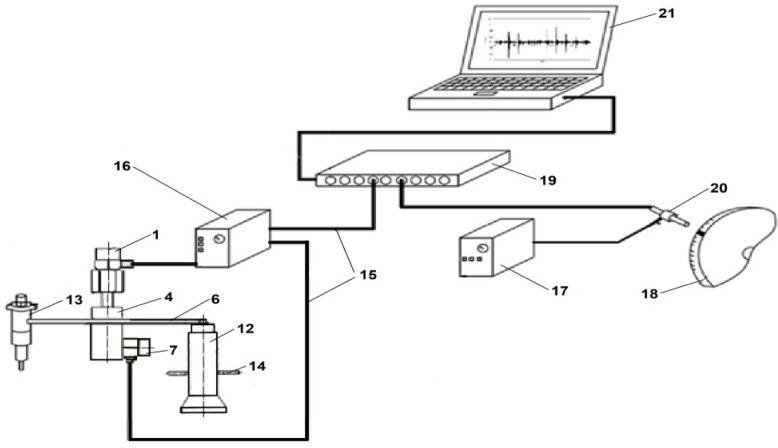
Schematic of the measurement setup [44]: 1, 7—vibration acceleration sensor, 4—holder, 6—injection pipe, 12—fuel injection pump, 13—injector, 14—fuel pump rack, 15—electrical connection wires, 16—signal amplifier, 17—power supply, 18—combustion engine flywheel, 19—terminal connector BNC, 20—crankshaft position sensor, and 21—notebook.

Figure 1 is a schematic of the components of the measuring device and the spatial configuration, which includes its connection to the injection system. It consists of a flywheel (18), charge amplifier (16), connection terminal (19), laptop computer (21), analog-to-digital card (22), and connecting cables (15). A unidirectional piezoelectric vibration acceleration sensor (7) is mounted between the injection pump (12) and the injector (13).

The analog-to-digital card is equipped with at least 10 V inputs for each cylinder being tested, plus one input for the crankshaft position signal, all sharing a common ground. The device has a piezoelectric unidirectional vibration acceleration sensor mounted on a holder, which is fixed parallel to the longitudinal axis of symmetry of the fuel injection pipe. The other two vibration acceleration sensors are mounted on a hexagonal-headed mounting bolt. The threaded stub bolt of the accelerometer mounting is screwed into a hole in the holder.

During the tests, the piezoelectric vibration acceleration sensors from Brüel & Kjær, Virum, Denmark were used. The measurement system also included a Brüel & Kjær preamplifier, Virum, Denmark. The specifications of the example accelerometer, type 4343, were as follows: a charge sensitivity of 10.12 pC/g, a voltage sensitivity of 8.61 mV/g, a capacitance (including cable) of 1176 pF, and a maximum transfer sensitivity at 30 Hz: 2.5% weight, 16.4 g, and an undamped natural frequency of 84 kHz. The TWD TOO12-16 ZP reflective photo-optical sensor TWT AUTOMATION, Warsaw, Poland included an M12 metal housing, a DC power supply of 10–30 V, and a load current of 150 mA.

A Brüel & Kjær, Virum, Denmark accelerometer calibrator was used to calibrate the vibration acceleration measurement system, allowing for the selection of the appropriate gain in the signal acquisition system. In the amplifier and matching circuit assemblies, the signals were amplified to match the sensors’ sensitivity and were then adapted to the analog-to-digital converter inputs. The reliability of the test facilities is described in previous work [6]. A control and steering system can be used to automate the diagnostic process. The device’s limitations include thermal loads and the risk of liquid exposure.

The device allows vibration processes in three directions, with the sensor mounted along the fuel injection pipe generally providing the strongest signal. The terminal connector enables simultaneous measurement of multiple signals from individual cylinders of an ICE. Advanced signal evaluation is possible through the visualization of raw signal waveforms and various modern signal analysis techniques can be applied.

### 2.3. Description of the Innovation

The presented innovation involves a testing stand and a diagnostic procedure for the fuel injection system in the piston SIEs, which uses an accelerometer and signal measurements. The goal here is to design a device that optimally diagnoses the injection system. The research was conducted both in a laboratory and under ship conditions. Technical data for the most commonly used combustion engines are presented in Table 1.

**Figure 2:**
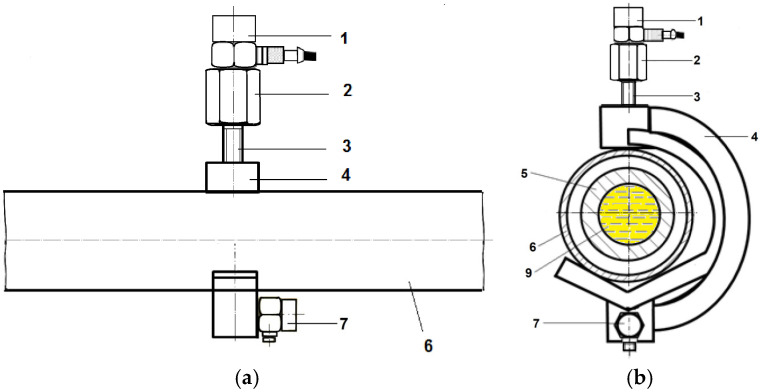
(**a**) Vibration acceleration sensors mounted vertically and longitudinally on the injection pipe and (**b**) cross-section of the injection fuel pipe with casing: 1—vibration acceleration sensor mounted vertically orthogonal to the fuel injection pipe, 2—head of the vibration acceleration sensor mounting screw, 3—threaded bolt of the vibration acceleration sensor mounting screw, 4—vibration acceleration sensor holder, 5—injection pipe, 6—shielding tube for the injection pipe, 7—vibration acceleration sensor mounted longitudinally along the injection pipe, and 9—fuel (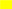).

The measuring system is characterized by piezoelectric unidirectional vibration acceleration sensors mounted on a holder, which is fastened to the fuel injection pipe (Figure 2a). The holder (4) has a *T*-shaped cross-section (Figure 2b) with two conical planes positioned at right angles. Figure 2a shows the shielding tube (6) and the injection pipe (5), with the holder (4) mounted vertically. The vibration acceleration sensors are positioned as follows: One sensor is mounted vertically on the hexagonal head of a screw (2) and the second accelerometer is mounted horizontally on the holder (4), along the fuel injection pipe (7). Figure 2b shows the cross-section of the shielding tube (6) and the injection pipe (5), which is filled with fuel (9), with the vibration acceleration sensors mounted vertically (1) and horizontally (7) on the holder (4).

The threaded bolt of the vibration acceleration sensor mounting screw is inserted into the hole in the holder. In the device, the holder covers the injection pipe or the casing of the injection pipe. The holder is secured with a threaded hexagonal head bolt, and a torque is applied using a torque wrench to ensure no plastic deformation occurs in the injection pipe or casing. The hexagonal head screw is made of low-alloy steel 45 and features a threaded hole along its axis, allowing for the vibration acceleration sensor to be securely attached.

Signals from the vibration acceleration sensors are transmitted through electrical connection cables and a signal amplifier to a terminal connector, which connects to an analog-to-digital card in the laptop computer. A crankshaft position sensor, in cooperation with a crankshaft marker (an adhesive aluminum foil strip) on the flywheel of the ICE, receives power from a feeder and is used to select the timing and the initiation of the signal acquisition. The data acquisition system consists of a device capable of storing large amounts of data in its operating memory, controlled via a laptop computer. Once saved, the data are analyzed using Excel 365 [Supplementary Materials], MATLAB 2022a, and additional software tools. To avoid sampling errors, the signals are low-pass filtered.

**Figure 3:**
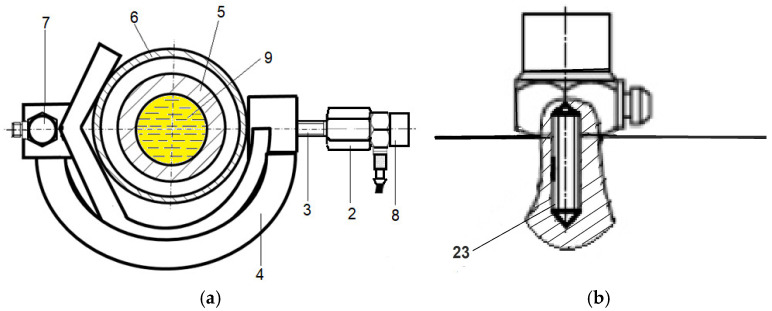
(**a**) Cross-section of the shielding tube and fuel-filled injection pipe (9) with accelerometers mounted horizontally and (**b**) a method for attaching the acceleration sensors to the holder using a threaded steel pin: 2—head of the screw securing the vibration acceleration sensor, 3—threaded bolt of the screw fastening the vibration acceleration sensor, 4—vibration acceleration sensor holder, 5—injection fuel pipe, 6—protective tube for the injection pipe, 7—accelerometer fastened in the direction along the injection pipe, 8—accelerometer fastened horizontally orthogonal on the injection pipe, 9—fuel (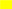), and 23—stud bolt.

Figure 3 shows a cross-section of the protective tube (6) and the high-pressure injection pipe (5) filled with fuel (9), with vibration acceleration sensors (7 and 8) mounted on a horizontally positioned holder (4). The accelerometer (8) was fastened perpendicularly to the injection pipe. The two accelerometers were mounted on a hexagonal head fastening screw (Figure 3b), with the sensors placed in a drilled hole with a thread that matches the threaded steel bolt of the sensor. Figure 3b shows how the vibration acceleration sensors (7, and 8) were fastened to the holder (4) using a threaded steel bolt (23). Difficulties in applying the method arose due to the flexible injection pipe covers, which could be displaced in AL20/24 engines.

**Figure 4:**
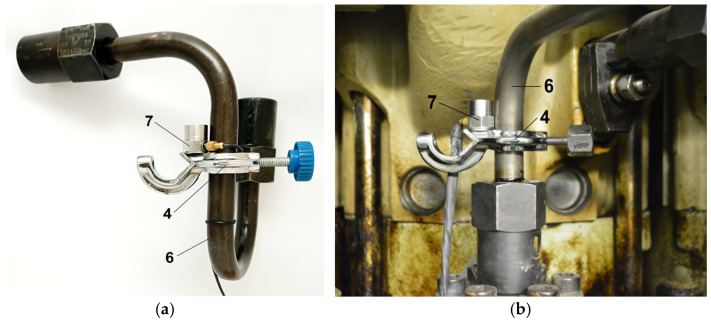
(**a**) Locations of the sensor holder on an example pipe and (**b**) view of the sensor location at the injection pump: 4—holder with sensors on the injection line, 6—fuel pressure pipe, and 7—vibration acceleration sensor.

**Figure 5:**
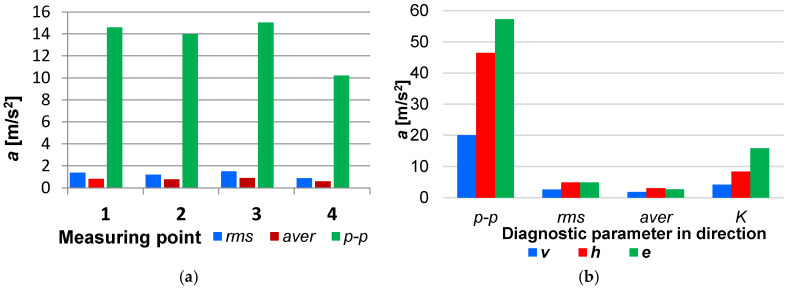
(**a**) Impact of the sensor mounting location on the amplitude estimates of the vibration acceleration signals *a* and (**b**) the effect of the signal processing direction on amplitude measurements and kurtosis: 1—at the injection pump, 2—before the bend, 3—between bends, and 4—at the stud for the fuel injector. In these graphs, *p*-*p* is the interpeak value, *rms* is the RMS value, *aver* is the mean value, *K* is the kurtosis, *v* is the vertical, *h* is the horizontal, and *e* is the longitudinal axis of the injection pipe.

Figure 4a illustrates the sensor location on an example fuel injection pipe and Figure 4b shows the mounting arrangement, which affects the values of the vibration acceleration signals (Figure 5a). The recommended sensor location is as close as possible to the injection pump to obtain the diagnostic parameter in the specified direction.

To determine whether the sensor location significantly affects the diagnostic parameters, the statistical hypothesis *H*_o_: ${\overset{\text{-}}{y}}_{1}={\overset{\text{-}}{y}}_{2}$, equality of two means in the populations of research results, was tested. However, the location presented in this manuscript proved to be optimal.

### 2.4. Procedure and Conditions for Testing the Innovative Solution

A measuring system for diagnosing the fuel injection system in the piston SIEs using vibration signal measurements, which is now patented, consists of the following components [44]: accelerometers (1, 7, and 8), electrical connecting cables (15), a signal amplifier (16), a power feeder (17), a terminal connector BNC (19), a photo-optical crankshaft position sensor (20), a portable notebook (21) with an analog-to-digital card (22), a signal acquisition and analysis system, and stud bolts (23) for mounting the vibration acceleration sensors.

A piezoelectric unidirectional vibration acceleration sensor (7) is mounted on a holder (4), positioned parallel to the longitudinal axis of symmetry of the fuel injection pipe. The holder (4) has a *T*-shaped cross-section with two conical planes located at right angles. The other two accelerometers (1 and 8) are mounted on a hexagonal head fastening screw (2), with the sensors (1 and 8) placed in a drilled hole with a thread that matches the threaded steel stud bolt (23) of the sensor. The threaded bolt of the accelerometer mounting screw is fastened into the hole in the holder.

The selected diagnostic parameters are compared with the predetermined limit values, and the tested injection system is the classified into one of two states on a technical state. The ability state refers to the full capability for the injection system and its ability to fulfill the entire load range of the SIE. This solution uses diagnostic measures that relate to various of the technical state features of the fuel injection system.

Since engine operating conditions, such as relative load, crankshaft speed, and thermal conditions, affect the diagnostic symptom values [46,47,48], it is recommended to select a relative engine load level [45] where changes in load do not significantly affect the error. The symptom measurements should be performed under repeatable conditions; the limit values are determined using these measurements. The latter are averaged and adjusted by plus or minus three times MEM, extended by the upper and lower tolerance intervals. An ability condition is assumed when between these determined lower and upper limit values, while disability conditions are outside these limits.

The measurements for assessing the technical condition of the injection equipment, made under the nonrepeatable values of rotational speed, fuel setting, and combustion engine or test bench thermal conditions, should be corrected to repeatable conditions. Plots of diagnostic parameter values were elaborated as a function of relative load, using a second-degree polynomial model in the form of *y* = *f*(x). According to this model, diagnostic parameter values can be calculated by inserting the expected engine load as variable *x*. For such cases, alongside correcting nonrepeatable conditions, the method for diagnosing piston ICEs during both the manufacturing and operating phases requires comparing the measured parameters with reference values. The interrelationships between the working process and the external conditions are complex [45]. Standard external conditions have been established for this purpose, for which the measured values are compared. According to ISO 3046-1:2002 (E) [49], the external conditions are assumed to be the following:

- A barometric pressure of 100,000 Pa;
- An air temperature of 25 °C;
- A relative humidity of 30%;
- A charge air coolant temperature of 25 °C.

These values are described in a reference book [5], as well as by Piotrowski and Witkowski [50]. The diagnostic method was carried out with a relative measurement error of ≤4%.

The correction factor is a function of the environmental and measured parameters under normal and operating conditions [45,50].

## 3. Research Results

**Figure 6:**
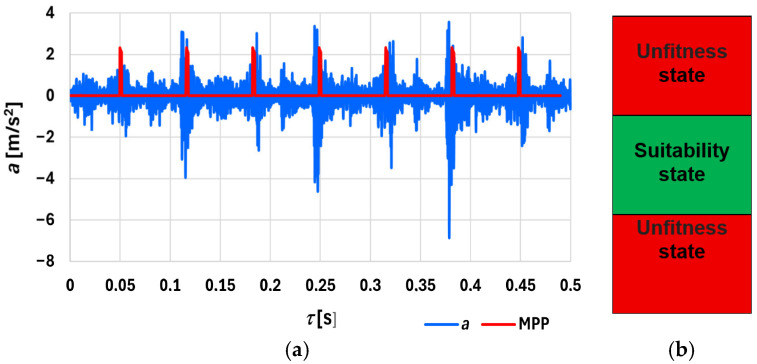
(**a**) Example time course of the vibration acceleration output *a* and the pulses corresponding to the bottom and top dead center of the piston (**b**) calculated tolerance fields: *τ* is time, *a* is the vibration acceleration signal, and MPP is the dead center of the piston.

**Figure 7:**
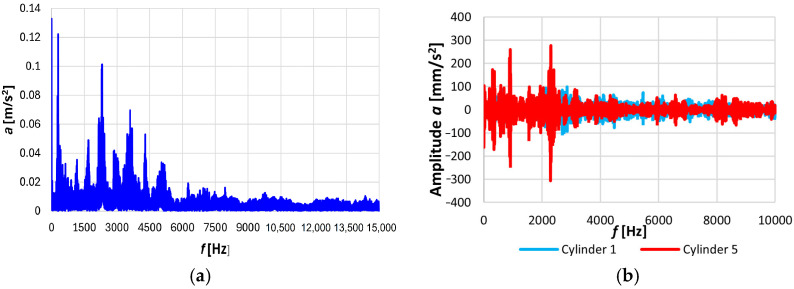
(**a**) Amplitude spectrum of the vibration acceleration signals *a* processed on a mount along the injection pipe of a 4L20 engine in the 0–15,000 Hz frequency range, and (**b**) the frequency transform scope of this signal for the ability state for the first nozzles and fractured nozzles (5).

**Figure 8:**
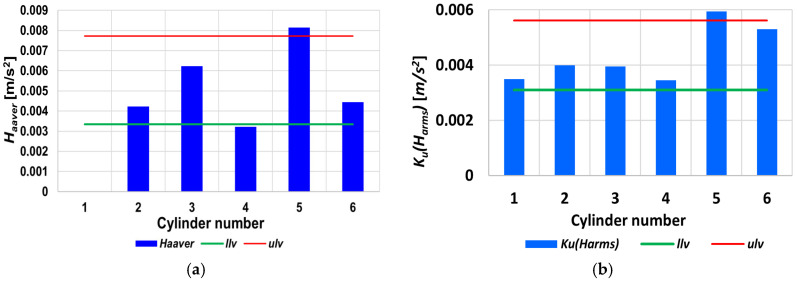
(**a**) Average values of the component amplitudes in the *H_aaver_* third-octave bands with a central frequency at 630 Hz (Supplementary Materials), which are proportional to the fuel injection frequency, and (**b**) the cumulative amplitude values from the first to the 21st third-octave band *K_u_* (*H_arms_*).

**Figure 9:**
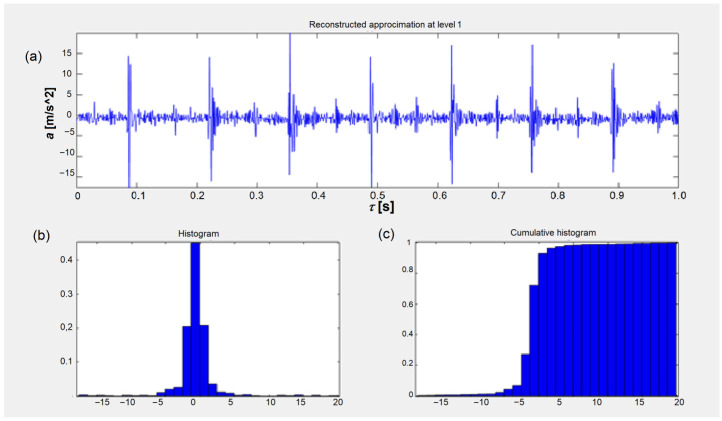
(**a**) Results of applying a statistical Haar wavelet approximation at the first level, (**b**) corresponding histogram and (**c**) cumulative histogram for the first approximation level: *a* is vibration acceleration signal and *τ* is time.

**Figure 10:**
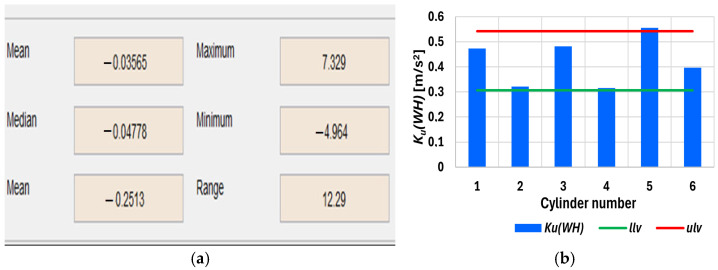
(**a**) Haar wavelet statistical parameter values of the vibration acceleration output for the injector and (**b**) the cumulative Haar wavelet approximation statistics *K_u_*(*WH*) for all the cylinders of the 6AL20 engine [Supplementary Materials].

The method was verified under conditions on a ship, where measurements exceeding the limit values (Figure 6b) confirmed damage to the injector nozzles. Advanced analyses of the proposed signals were conducted using a stand or bed, and a procedure for diagnosing the fuel injection system in the piston SIEs was used. Vibration acceleration signal measures were applied in the following domains: time (Figure 6), amplitude (Figure 5), frequency (Figure 7 and Figure 8), and wavelet transform (Figure 9 and Figure 10).

The optimization criterion was the maximum signal energy at different locations of the vibration acceleration sensor. Figure 5a illustrates the effect of the sensor mounting location on the amplitude estimates of the vibration acceleration signal time histories. Figure 5b demonstrates the impact of the signal measurement direction along the injection pipe. On average, this was 2.5 times the vertical direction and 1.25 times the horizontal direction for the vibration acceleration signals.

Figure 5a shows that the closer the sensor is to the injection pump, the higher the amplitude estimates, which is due to the pressure losses and the dissipation of vibration energy. However, at the pump itself, the connector stiffens the hose, which affects the measurements. Figure 5b shows that, for all the diagnostic parameters, except for the average and *rms* values, the highest values occur for vibrations measured along the injection fuel pipe (amplitude estimates were up to 2.86 times higher than in the vertical and horizontal directions). This is because fuel waves travel along the injection pipe.

In the fuel injection process, phenomena related to fuel flow and injector component behavior can be distinguished. Fuel flow through the injection pump and injector, as well as within the pump, causes: natural vibrations of the fuel column in the pipe, turbulence resulting from fuel pipe resistance, and hydraulic shocks. The frequency of natural vibrations of the fuel volume caused by a pressure wave in the injection pump-injector path is as follows:

(4) $$f_{sp}=\frac{u}{2l}$$

where *u*—speed of the pressure wave caused by piston movement, and *l*—length of the injection pipe. The vibrations during fuel injection have characteristic frequencies, which were calculated to identify the components in the acceleration spectra that contribute the most power along the injection pipe. The dynamics of the mechanical components of the injection subsystem, in which the equation describing the motion of the nozzle needle and fuel pump of moving elements during fuel injection, was derived [2]. Figure 6a shows an example time waveform, *τ*, of the vibration acceleration signal, *a*, with three fuel injection pulses, along with the bottom and top dead center (MPP) markers. Figure 6a illustrates that the fuel injection pulses occur at the same position relative to the piston dead center and are asymmetrical on both sides. It is recommended that the time window contains signals from at least three cycles, so that signal captures include sufficient data for analysis. Due to the unique nature of the fuel injection, diagnostic measures should be averaged. To determine the number of averages, statistical hypotheses *H*_o_ about the equality of two means were verified, showing that 16 is sufficient.

The raw time course, *τ*, of the periodic vibration acceleration signal, *a*, with three regular fuel injection pulses is employed. The measurement path also includes elements used to trigger the start of the signal acquisition and recording, as well as the time selection chosen with regard to the crankshaft or camshaft position sensor.

Figure 7a shows the frequency, *f*, spectrum of the vibration acceleration signal in the 0–15 kHz range. This spectrum displays the amplitude values for individual frequencies, including those associated with the fuel injection frequency. Figure 7b presents the frequency transform scope of the signal, which is used to identify new and useful diagnostic symptoms [Supplementary Materials].

The frequency transform scope can be used to read the frequency at the cursor position, the amplitude value from the vertical axis, the most significant negative value achieved, the highest value reached by the selected channel, the peak frequency of the transformation spectrum, and the power calculation. The determined power values were used to identify the optimal horizontal mounting of the sensor along the injection pipe. The RMS value of the vibration acceleration is an estimate of the signal power. Unfortunately, the power value for cylinder No. 6, in an ability state, is greater than that of cylinder No. 5, which has a developing fracture. As a result, this method of signal analysis was abandoned.

The suitable frequency (in Hz) in the third-octave bands is determined using the following formula [2,14,50]:

(5) $$f_{av}=\frac{n_{o}}{60}100,$$

where *f_av_* is the center frequency of the third-octave band and *n_o_* is the crankshaft rotational speed.

When examining the relationships between the individual diagnostic parameters and the technical state, the results were not always clear-cut (Figure 8a). However, characteristic frequencies associated with typical processes occurring in ICEs were calculated. This is due to the non-repetitive processes in internal combustion engines, changes in external conditions, and disturbances. Therefore, their accumulations were used for diagnosing fuel injectors. They make it easier to analyze sums of independent random variables, since the cumulative sums are equal to the sum of the cumulants of the components. For example, for the RMS values of the amplitudes in the vibration acceleration spectra across the third-octave bands, the accumulations of amplitude values from the first to the 21st third-octave band, which the authors have developed (Figure 8b), can be found using the following:

(6) $$K_{u}\left(H_{arms}\right)=\sqrt[{21}]{H_{arms1}\times H_{arms2}\times H_{arms3}\times \ldots \times H_{arms21}},$$

where $H_{arms1}$ = 12.5 Hz (the revolution frequency of the combustion engine crankshaft *f_o_*), …, $H_{arms21}$ = 1250 Hz (*f_o_* × 100).

Figure 8 shows the upper limit and indicates a disability state since it is exceeded. Additionally, the accumulation of the amplitude values across the third-octave bands has increased the differences between the ability and the faulty state of symptoms. Figure 9a depicts the result of applying an example statistical measurement of the Haar wavelet to the approximation at the first level. Figure 9b and Figure 9c show the corresponding histogram and cumulative histogram for the first level of approximation, respectively.

The equation for the Haar wavelet approximation, in the form of a function *f*(x), can be described as follows [51]:

(7) $$f(x)\approx \sum_{i=1}^{2^{M}}\;c_{i}h_{i}(x)=c^{T}H_{2^{M}}(x),$$

where $\sum_{i=1}^{2^{M}}\;c_{i}h_{i}\left(x\right)\;$ is a finite sum, $c_{i}$ is the Haar coefficients, *h_i_*(*x*) is the Haar functions, $H_{2^{M}}(x)=[h_{1}\left(x\right),h_{2}\left(x\right),\ldots ,h_{2^{M}}\left(x\right){]}^{T}\;is\;the\;Haar\;function\;vector,\;and\;c^{T}=\left[c_{1},c_{2},\ldots ,c_{2^{M}}\right]$ is the coefficient vector. Figure 10a displays the results for the statistical parameters of the Haar wavelet for the example approximation level. These statistical measures include the average, median, maximum, minimum, range, mean-square error, median of absolute error, and average absolute deviation. The maximum norm at levels 1 and 2 has been omitted due to its low correlation with the state.

When searching for the optimal diagnostic symptoms, the accumulation of the selected Haar wavelet approximation statistics for all the cylinders of the 6AL20 medium-speed engine, along with the lower (*llv*) and upper (*ulv*) limit values, was used; these values are shown in Figure 10b and expressed using the following expression:

(8) $$K_{u}\left(WH\right)=\sqrt[{9}]{\begin{array}{c} Mean\times Median\times Mode\times Maximum\times Minimum\times Range\times Standarddev\times Median\;abs.\;dev.\\ \times Mean\;abs.\;dev.\;\; \end{array}}$$

Figure 10b shows that, for injector No. 5, the lower limit was exceeded due to the development of damage. After approximately 250 working hours, this SIE was stopped due to the high exhaust gas temperature. A fragment of injector nozzle body No. 5 was found to have broken off.

The method was verified by measuring the acceleration signals before periodic maintenance of the ICE. After dismantling the 4L20 engine, the technical state features of the injectors and nozzles were measured, and correlations with diagnostic parameters were analyzed using the following:

*P_a_* = *f*(*α*, *u_n_*, *i_o_*, *F_fa_*, *h_max_*, *L_a_*, *P_o_*, *q_f_*), (9)

where *α* is the sealing cone angle, *u_n_* is the speed of the needle protruding from the nozzle body, *i_o_* is the number of open holes, *F_fa_* is the average friction force between the body and the nozzle needle, *h_max_* is the maximum needle stroke, *L_a_* is the average diameter clearance between the mating surfaces, *P_o_* is the injector opening pressure, and *q_f_* is the air volume flow flux through the nozzle.

Multiple regression was used to determine the significance of the impact of technical state features on diagnostic parameters, which yields the following expression for the interpeak value:

*a*_*p*-*p*_ = −14.1577 + 16.4017*α* + 16.2215*L_a_* ± 0.1135 (10)

The model was statistically significant based on analyses conducted in Statistica 13.3. software, as indicated by an *F*-test with *p* < 0.04525. The coefficient of determination was very high (*R*^2^ = 0.9939) and all the regression coefficients were statistically significant.

**Figure 11:**
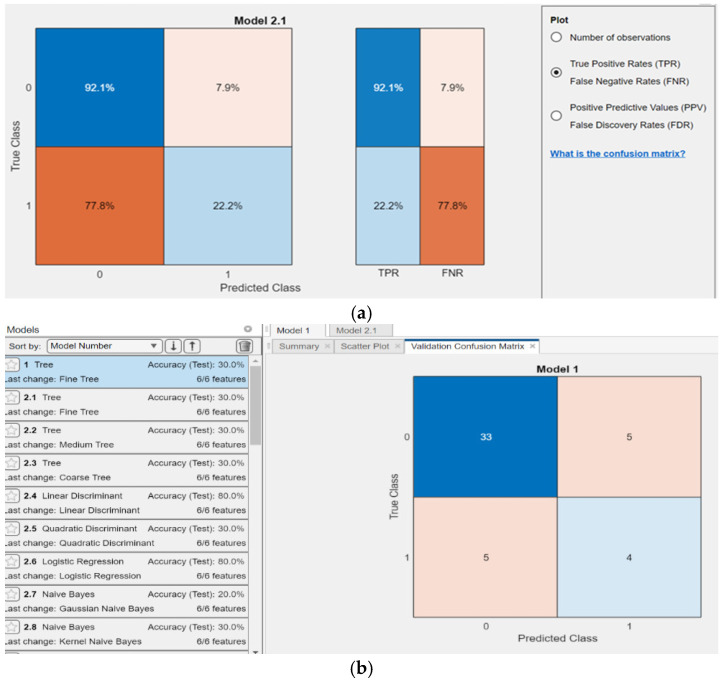
Accuracy test results for classifying the diagnostic vibration parameters: (**a**) applied according to model 2.1 and (**b**) exemplary results with poor testing quality for 462 samples.

The diagnostic parameters of the vibration acceleration signal were selected and relevant symptoms were identified. The assessment was based on a matrix for accurately diagnosing fuel injection systems with developing damage. Artificial neural networks and machine learning in active experiments [52] were also used for damage classification, as in passive experiments (Figure 11). The architecture of the procedure stages, from signal acquisition from the internal combustion engine to the development of a classification and recognition model, is presented in publication [2]. For effective neural network learning, the more connections there are in the network, the more training cases are needed. The number of training cases was set at a minimum of 60. For a set of 82 diagnostic parameter values from the training data, determined across six cylinders of the tested engine, the best networks were selected. The highest correlation statistics for the test and validation samples were used as the criterion for selecting the best networks. The classification learning tool in MATLAB 2022a and Statistica 13.3 achieved a validation accuracy of 85.1% for supporting vector machine neural networks and a testing accuracy of over 80.9% for many models. As shown in Figure 11b, some models demonstrated poor testing quality.

The meaning of the colors in the confusion matrix for the classification learner is as follows: dark blue (diagonal) indicates true positives or True Negatives. Lighter shades/white (outside the diagonal) indicate classification errors. They represent False Positives or False Negatives. The cream-colored diagonal represents incorrectly classified data. Brown/orange shades indicate classification errors. 

A matrix for accurately identifying developing damage was created using binary records (Table 2). It was assumed that the network correctly classified the state when the overall testing accuracy was at least ≥70.0%. When the diagnostic parameter detected developing damage and the classification learner correctly classified the technical state, the symptom was considered a valid and useful indicator. The quality of the network as a classifier was evaluated using the receiver operating characteristic (ROC), which plots cumulative specificity (false positives) on the *x*-axis and sensitivity (true positives) on the *y*-axis [52].

The tabular assessment of the usefulness of the selected diagnostic parameters in various contexts helps to avoid subjective errors by the diagnostician. Existing solutions in the literature often describe the use of vibration magnitudes but fail to specify the signals, parameters, test conditions, or limit values above or below the threshold. In contrast, this method specifies the test conditions and the conversion of diagnostic parameters into normal conditions in accordance with ISO standards. During the analysis of the table data, pairs of checks (*C_p_*_1_, *C_p_*_2_) were constructed, where 0-1 and 0-0 indicate no usefulness (0) and 1-1 signifies usefulness (1).

For the obtained results, the probability of correct diagnosis *Q_d_* is calculated using the following formula:

(11) $$Q_{d}=\frac{n_{z}}{n_{n}},$$

where *n_z_* is the number of states of the injection system that are consistent with the state recorded during operation, and *n_n_* is the number of states of the injection system. A tabular evaluation of the ability of the selected diagnostic measures across various possibilities shows that *Q_d_* = 0.467 of them proved useful for detecting developing damage. This is a measure of the validity of the selection of the diagnostic parameters included. These included interpeak values in the amplitude domain, cumulants for the amplitude measures, mean amplitudes in the third-octave bands, cumulants of the effective amplitudes in the third-octave bands, maxima of the Haar wavelet statistics, and cumulants of the Haar wavelet statistics.

**Figure 12:**
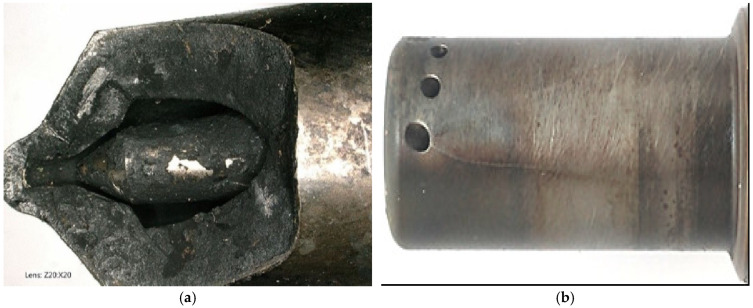
(**a**) Breakage of a fragment of the classical injector nozzle. (**b**) Longitudinal crack from the spray hole of an electronically controlled engine injector nozzle MAN 032050070 0036 HJ.

Injector nozzle 159 × 9 × 0.25R × N0.5 fractures are a common issue in both conventional marine combustion engines (Figure 12a) and electronically controlled main engines (Figure 12b). Despite advances, nozzle fractures continue to occur, even in modern S50ME-B9 electronically controlled main drive engines. This highlights the ongoing need for effective diagnostics to identify the causes of damage. Four nozzles, with an average work time of 1163 h, were found to be fractured.

During these tests, slight changes in the diagnostic symptom values corresponding to the initial development of damage were observed (Figure 12b). Identifying the faulty state aligns with reductions in fuel consumption, toxic emissions released into the atmosphere, and engine noise.

A single crack growth equation for injector nozzles is not available in the literature because it is a complex process modeled by fracture mechanics equations, which are often analyzed using computational methods such as the finite element method. Descriptions of fatigue cracking in steel are provided by Radkowski [1] and Duraipandi et al. [53]. Here, their descriptions have been adapted to injector nozzles. This relationship considers a range of degradation growths in the injector valve nozzles and diagnostics [2]. An attempt was therefore made to formulate an equation based on indicated factors in the literature and our own research that influences the initiation and propagation of injector nozzle fracture *C_d_*, which results in the following:

*C_d_* = *f*(*M_t_*, *Ge*, *M_s_*, *S_s_*, *S_w_*, *P_f_*, *P_c_*, *T_w_*, *E_L_*, *n_e_*, *n_c_*, *W_s_*), (12)

where *M_t_* is the injector material, *G_e_* is the injector nozzle geometry, *M_s_* is the assembly stresses, *S_w_* is the working stresses, *S_s_* is the spring stiffness, *P_f_* is the fuel pressure time waveform, *P_c_* is the cylinder pressure time waveform, *T_w_* is the temperature profile, *E_L_* is the ICE load, *n_e_* and *n_c_* are the crankshaft and camshaft speeds, respectively, and *W_s_* is the total wear. The authors measured all quantities in Equation (12) except for the assembly stresses *M_s_* and the working stresses *S_w_*; these are planned for future research. The total relative error of the measured vibration acceleration parameter, $\bar{\sigma_{t}}\left(P_{a}\right)$, is estimated using the following:

(13) $$\bar{\sigma_{t}}\left(P_{a}\right)=\pm \;\sqrt{\frac{1}{3}{\left[\bar{\sigma }(P_{i})\right]}^{2}+{\left[\bar{\delta }(P_{i})\right]}^{2}+{\frac{1}{3}\left[\bar{\sigma }(P_{u})\right]}^{2}+{\left[\bar{\delta }(P_{u})\right]}^{2}},$$

where $\bar{\sigma }\left(P_{i}\right)$ is the relative random error of the fuel injection parameter, $\bar{\delta }(P_{i})$ is the systematic random relative error of the fuel injection parameter, $\bar{\sigma }\left(P_{u}\right)$ is the relative random error of the vibration acceleration measurement track, and $\bar{\delta }(P_{u})$ is the systematic relative error of the vibration acceleration measurement track.

The relative maximum error of the signal processing for the measured vibration acceleration section is calculated via the following:

(14) $$\bar{\sigma }\left(P_{u}\right)=\pm 100\%\sqrt{{\left(\frac{{∆}_{bs}}{a_{ts}-a_{bs}}\right)}^{2}+{\left(\frac{{∆}_{bp}}{a_{tp}-a_{bp}}\right)}^{2}+{\left(\frac{{∆}_{bd}}{AD}\right)}^{2}\;\;},$$

where ${∆}_{bs}$ represents the limiting absolute error of the accelerometer, ${∆}_{bp}$ denotes the limiting permissible absolute error of the power supply,$\;a_{ts}-a_{bs}$ is the measuring range of the accelerometer, $a_{tp}-a_{bp}$ is the measuring range of the signal amplifier, ${∆}_{bd}$ is the absolute error of the analog-to-digital converter, and *AD* is the number of bits of the analog-to-digital converter.

Obtaining quantitative data for Formula (9) is not straightforward, but it is possible even if the manufacturer conceals the material specifications. The material of the nozzles can be determined using a modern microscope.

## 4. Discussion

Based on current knowledge and technology, vibration acceleration sensors can be mounted invasively using threaded connections [41] or, more effectively, with noninvasive holders and methods [42]. The proposed shape of the holder and the placement of the vibration acceleration sensors enable highly effective diagnostics that were not possible with other grip designs or various fuel injection pipe and casing solutions. The modified holder features two conical planes arranged in a *V*-shape at right angles, allowing it to adapt to injection pipes of different diameters. It is made of electrostatically coated material to protect against interference. The *T*-shaped cross-section of the holder increases its rigidity. This design had an unexpected effect, i.e., it caused no plastic deformation of the injection line or the injection pipe casing, which is a requirement for noninvasive diagnostics. The innovative features of the holder—namely, its *T*-shape, material, and optimal placement of the vibration acceleration sensors—enable highly effective diagnostics that were not achievable with previous designs. The author of this article tested various holder designs and sensor mounting locations; the presented configuration was found to be optimal.

A surprising finding was that the vibration acceleration signals received along the injection pipe had the strongest magnitude, with amplitude estimates up to three times higher than for the vertical and horizontal directions (for example, determined by the calculated RMS). This was unexpected, since one might have assumed that a perpendicular mounting relative to the injection pipe axis would produce stronger signals. It is noteworthy that this type of sensor mounting is not found in the current literature, which suggests that specialists have not previously considered it useful for diagnostic measurements.

For existing solutions, the comparison of the measured signal with a reference signal is typically performed manually, organoleptically, or based on the diagnostician’s knowledge and experience [13,54]. In contrast, this article presents an automatic, strictly defined procedure for performing such comparisons. Similar solutions in the literature describe the use of vibration magnitudes but do not specify the signal parameters, test conditions, or limit values (either smaller or larger than a threshold) [54]. In this study, the limit values are determined based on the average symptom value for the ability state plus or minus three times the MEM, a method not found in the literature, using an expanded uncertainty at a 99.7% probability. Another distinction is the use of three multiples instead of two or four, which affects accuracy when determining the limit values.

Furthermore, this method offers a novel two-state classification of the fuel injection system based on its current condition. The selected diagnostic symptoms showed a very close correlation with the technical state features (>0.900). They were effective in detecting developing damage, with an accurate diagnosis probability of *Q_d_* = 0.467.

## 5. Conclusions

The presented method for diagnosing a fuel injection system in the piston ICEs was verified under normal ship operation conditions. Namely, exceeding the limit values following the vibration acceleration signal measurements meant a faulty state for the fuel injection, which was confirmed by the observed damage to the injector nozzle and the relationships with technical state features. Advanced studies for the proposed signals were conducted using a device and a method for diagnosing injection equipment in piston ICEs, based on vibration acceleration signal parameters in the time, frequency, amplitude, and time–frequency domains.

An example time course for a vibration acceleration signal, including multiple fuel injection pulses, was also presented; it indicated the bottom and top dead centers of the piston. The fuel injection pulses occur at the same position relative to the piston’s dead center but are asymmetrical on both sides. It is recommended that the signal capture time window includes data from at least three cycles. Due to the unique nature of fuel injections, the diagnostic measurements should be averaged.

The spectrum of the output signal can be used to determine the amplitude values for individual frequencies, including those relating to the fuel injection frequency. The measurement path may also include components used to trigger the start of signal acquisition and recording, as well as the domains for time selection using a crankshaft or timing position sensor. The diagnostic parameters of the vibration acceleration signal were reduced in number, and the relevant symptoms were selected. The basis for evaluating these parameters was a matrix for identified faults, along with the correct classifications made by artificial neural networks. The usefulness of the diagnostic symptoms was considered in terms of a binary identification and classification (1,1). A frequency transform scope, which has not previously appeared in the literature, was tested; however, its use was not recommended.

Selected vibration acceleration symptoms in the amplitude domain, frequencies in third-octave bands, and some Haar wavelet statistics were found to be particularly sensitive to developing damage. The quality of damage recognition was improved by calculating the cumulants from multiple similar diagnostic parameters.

The estimated limit values helped select the diagnostic symptoms by confirming variations for the technical states across the different domains of the diagnostic signal analysis. The measurements of vibration acceleration signals in selected test domains were used to detect damage. A noteworthy contribution of this study is the identification of lower and upper limits that distinguish between two different technical states relating to developing damage. To date, no one has applied the estimation of limit values in diagnostics using expanded uncertainty with a probability of 0.977MEM; instead, they mostly rely on the standard deviation. The limitation is that the limit values are not measured after the engine has been manufactured or maintained. When the vibration acceleration limits for internal combustion engines after repairs were determined, the design characteristics of the injection apparatus and its technical condition were not available in a passive experiment on a ship.

Conclusions were drawn from the resulting secondary engine damage, and design and operating recommendations were formulated. Clearly, innovative solutions for detecting damage to fuel injectors should be implemented both during manufacturing and under operational conditions.

## 6. Patents

The device and method for diagnosing the fuel injection system of reciprocating internal combustion engines using vibration acceleration signal symptoms correspond to Patent No. 246619 at the Patent Office of the Republic of Poland, Warsaw, 17 February 2025 [44].

## Figures and Tables

**Table 1:** Technical data for the most frequently tested marine combustion engines.

Engine Type	6AL20/24	L16/24	L20D2
Number of cylinders	6	6; 7	4; 6
Engine speed [rpm]	750; 900	1000; 1200	900
Mean piston speed [m/s]	6; 7.2	8; 9.6	8.4
Cylinder diameter [mm]	200	160	200
Piston stroke [mm]	240	240	280
Stroke volume [cm^3^]	7540	6824	8796
Normal output [kW]	72.5; 82	90; 110	170
Compression ratio	1:12.7	15.2:1	16:1
Brake mean effective pressure [MPa]	1.48	2.24/2.58	2.44
Injector opening pressure [MPa]	24.5; 40	45	45
Max. combustion pressure [MPa]	12.0; 12.5	17.0; 18.0	16.8

**Table 2:** Matrix for recognizing developing damage for selected diagnostic symptoms.

Diagnostic Parameter	Nozzle Crack	Neural Networks Classification	Usefulness
*a_aver_*	0	1	0
*a_rms_*	0	0	0
*a*_*p*-*p*_	1	1	1
*K*	0	0	0
*K_u_*(*a_aver_*, *a_rms_*, *a_rms_*)	1	1	1
$H_{aaver18}$, $H_{aaver19}$	1	1	1
$H_{arms18},\;H_{arms19}$	0	0	0
$H_{aaver21,\;H_{aaver22\;\;\;}}$	0	1	0
$H_{arms21}$, $H_{arms22}$	0	1	0
$K_{u}\left(H_{arms}\right)$	1	1	1
*FTS_a_*	0	1	0
*W_HMax_*	1	1	1
*W_HR_*	0	1	0
*K_u_*(*WH*)	1	1	1

Note. 0 denotes the developing damage undiagnosed, 1 signifies the developing damage diagnosed, *FTS_a_* is the frequency transform scope, *W_HMax_* is the Haar wavelet maximum, *WHR* is the Haar wavelet range, and *K_u_*(*WH*) is the cumulative Haar wavelet approximation statistics.

## Data Availability

Data are contained within the article and supplementary materials.

## Supplementary Materials

The following supporting information can be downloaded at: https://www.mdpi.com/article/10.3390/s26061981/s1, Figures Excel, *H_aaver_* 630 Hz, *K_u_*(*WH*), frequency transform scope.

## Abbreviations

The following abbreviations are used in this manuscript:

*a*	vibration acceleration signal
*a_aver_*	average value of the vibration acceleration signal
*a*_*p*-*p*_	interpeak value of the vibration acceleration signal
*a_rms_*	RMS value of the vibration acceleration signal
*f*	frequency
*f_o_*	rotational frequency of the engine crankshaft
*H_eaver_*	average amplitude value in the third-octave band of the acceleration signal
*H_arms_*	RMS amplitude in the third octave of the acceleration signal
ICE	internal combustion engine
*K*	kurtosis
*K_u_*	cumulative value
*K_u_*(*a_aver_*, *a*_*p*-*p*_, *a_rms_*)	cumulative of the average value of vibration, interpeak value, and root-mean-square value of vibration acceleration signal
$K_{u}\left(H_{arms}\right)$	cumulations of amplitude values from the first to the 21st third octave
*K_u_*(*WH*)	cumulative Haar wavelet approximation statistics
*lev*	lower limit value
*ulv*	upper limit value
*P_a_*	diagnostic parameter
*p*-*p*	interpeak value
*rms*	root-mean-square (rms) value
SIE	self-ignition engine
*Wh_am_*	maximum value of the Haar wavelet
*σ**_s_*	mean-square error of the arithmetic average
*τ*	time
